# LAPAROSCOPIC SLEEVE GASTRECTOMY VERSUS LAPAROSCOPIC ROUX-EN-Y GASTRIC BYPASS FOR WEIGHT LOSS IN OBESE PATIENTS: WHICH IS MORE EFFECTIVE? A SYSTEMATIC REVIEW AND META-ANALYSIS

**DOI:** 10.1590/0102-672020230064e1782

**Published:** 2023-12-08

**Authors:** Laura GARCÍA-HONORES, Jose CABALLERO-ALVARADO, Alexander BUSTAMANTE-CABREJOS, Katherine LOZANO-PERALTA, Carlos ZAVALETA-CORVERA

**Affiliations:** 1 Antenor Orrego Private University, School of Medicine – Trujillo, La Libertad, Peru;; 2 Regional Hospital of Trujillo, Department of surgery – Trujillo, La Libertad, Peru;; 3 Alta Complejidad Virgen de la Puerta Hospital, Department of surgery – Trujillo, La Libertad, Peru.

**Keywords:** Morbid Obesity, Bariatric surgery, Gastrectomy, Gastric Bypass, Efficacy, Obesidade Mórbida, Cirurgia Bariátrica, Gastrectomia, Derivação Gástrica, Eficácia

## Abstract

**BACKGROUND:**

Bariatric surgery is the most effective option to reduce weight in morbid obesity patients. The techniques most employed are the restrictive surgery laparoscopic sleeve gastrectomy (LSG), surgical procedures of intestinal malabsorption, and both types (restrictive and intestinal malabsorption) such as the Roux-en-Y laparoscopic gastric bypass (RYLGB).

**AIMS:**

To determine if LSG is more effective than RYLGB for weight loss.

**METHODS:**

A systematic review and meta-analysis was carried out, including five clinical trials and sixteen cohorts comparing LSG versus RYLGB in weight loss and secondary outcomes: resolution of comorbidities, postoperative complications, operative time, hospital stay, and improvement in quality of life.

**RESULTS:**

Excess weight loss was 10.2% (mean difference [MD] 10.2; 95%CI -10.14; -9.90) higher in patients undergoing LSG than in patients submitted to RYLGB. Diabetes mellitus type 2 was resolved in 17% (relative risk [RR] 0.83; 95%CI 0.77–0.90) of cases, more significantly after LSG, arterial hypertension in 23% (RR 0.77; 95%CI 0.69–0.84), and dyslipidemia in 17% (RR 0.83; 95%CI 0.77–0.90). Postoperative complications were 73% higher in patients undergoing RYLGB (MD 0.73; 95%CI 0.63–0.83). The operative time was 35.76 minutes shorter in the LSG (MD -35.76; 95%CI -37.28; -34.24). Finally, the quality of life improved more in patients operated by LSG (MD 0.37; 95%CI -0.48; -0.26).

**CONCLUSIONS:**

The study demonstrated that LSG could be more effective than RYLGB in reducing the percentage of excess weight, comorbidities, postoperative complications, operative time, hospital stay, and in improving quality of life.

## RESUMO

**RACIONAL:**

A cirurgia bariátrica é a opção mais eficaz para reduzir o peso em pacientes com obesidade mórbida. Três técnicas são mais empregadas: gastrectomia vertical laparoscópica (LSG), procedimentos cirúrgicos de má absorção intestinal e ambos os tipos, má absorção intestinal e restritiva, e bypass laparoscópico em Y-Roux (RYLGB).

**OBJETIVOS:**

Determinar a eficácia da LSG versus RYLGB na perda de peso.

**MÉTODOS:**

Uma revisão sistemática e meta-análise foi realizada incluindo cinco ensaios clínicos e dezesseis coortes comparando LSG versus RYLGB na perda de peso e resultados secundários, resolução de comorbidades, complicações pós-operatórias, tempo operatório, permanência hospitalar e melhora da qualidade de vida.

**RESULTADOS:**

A perda de excesso de peso foi 10,2% (diferença média (MD) 10,2; IC95% -10,14; -9,90) maior em pacientes submetidos a LSG do que em pacientes operados com RYLGB. Diabetes mellitus tipo 2 foi resolvido em 17% (risco relativo (RR) 0,83; IC95% 0,77–0,90), hipertensão arterial em 23% (RR 0,77; IC95% 0,69–0,84), dislipidemia em 17% (RR 0,83; IC95% 0,77–0,90), mais significativos após LSG. As complicações pós-operatórias foram 73% maiores em pacientes submetidos a RYLGB (MD 0,73; IC95% 0,63–0,83). O tempo operatório foi 35,76 minutos menor na LSG (MD -35,76; IC95% -37,28; -34,24). Finalmente, a qualidade de vida melhorou mais no LSG (MD 0,37; IC95% -0,48; -0,26).

**CONCLUSÕES:**

LSG é muito eficaz na redução do excesso de peso, comorbidades, complicações pós-operatórias, tempo e melhora na qualidade de vida.

## INTRODUCTION

The World Health Organization (WHO) reports that the prevalence of obesity is increasing. Currently, 13% of the world’s adult population is obese, with a prevalence of 11% in men and 15% in women. This has made obesity a major global health problem^24,38^.

Excessive body fat increases the risk of complications in obese people, reducing their quality of life. The risk of having diabetes mellitus type 2 (DM2) increases proportionally to the rise in body mass index (BMI), being more pronounced in those above 30 kg/m^20,37^. There is also a directly proportional relationship between BMI value and high blood pressure values, so the prevalence rate of arterial hypertension (AH) in obese people is double that in thin patients^28^.

Bariatric surgery is the most effective therapeutic option^11^ for obese people. The conditions for choosing bariatric surgery, according to the consensus of the National Institute of Health (NIH) and the American Society for Metabolic and Bariatric Surgery (ASMBS), are BMI ≥40 kg/m^2^, or ≥35 kg/m^2^ with comorbidities and previously attempted weight loss for at least 6 months^2^. Three types of bariatric surgery are used: gastric restriction surgeries such as LSG, surgical procedures of intestinal malabsorption, and both restrictive and intestinal malabsorption type such as RYLGB^9,22^.

We conducted a systematic review of the literature and meta-analysis in order to compare weight loss between both surgeries in patients with morbid obesity. Besides, we evaluated the presence of complications, resolution of comorbidities, improvement in quality of life, and time of surgery and hospital stay as secondary outcomes.

## METHODS

The recommendations of Preferred Reporting Items for Systematic Reviews and Meta-Analyses (PRISMA) were taken into account to carry out this systematic review and meta-analysis.

Inclusion criteria

The predefined selection criteria for the present research were original articles from randomized clinical trials and observational cohort studies, published in a database, in English and/or Spanish, comparing the efficacy of RYLGB versus LSG in weight loss.

Exclusion criteria

All those articles that presented any of the following characteristics were excluded:

- Descriptive primary studies and case-control studies.
- Studies that have not yet been completed or whose results have not been published.
- Primary study articles in a language other than English or Spanish.

### Outcomes

The primary outcome is the percentage of excess weight loss. The secondary outcomes were the resolution of comorbidities through the normalization of laboratory tests, interruption of treatment, or some other piece of evidence that proves the resolution of AH, DM2, and dyslipidemia (DLP). Postoperative complications were also included as a secondary outcome, classifying them as major, minor, early, or late. Other secondary outcomes were the operative time of bariatric surgery expressed in minutes, hospital stay after bariatric surgery indicated in days, and improvement in quality of life measured through different rating scales.

### Literature search strategy

The advanced search was carried out in the following databases of medical literature data: PubMed, Web of Science, Scopus, Cochrane, Ovid Medline, MedRxiv, bioRxiv, and ClinicalTrials using an advanced search strategy.

### Study selection

Items were unloaded and stored in the Rayyan tool, in order to start the screening phase^37^. After the removal of duplicate articles, two reviewers independently revised the title, abstract, and content of each article. After resolving the conflicts and reaching an agreement, the relevant articles were selected, important data was extracted from full text and meta-analyzed.

### Data collection

The following data were extracted from the included studies: name of the first author, year of publication, country in which the study was conducted, number of patients included in the study, number of patients who underwent each surgery, and outcome results. The extracted data was stored in Microsoft Excel program.

### Assessment for risk of bias

Clinical trial studies were assessed for risk of bias using the Cochrane risk-of-bias tool. Observational cohort studies were assessed for risk of bias using the Newcastle-Ottawa Scale (NOS). Each item in each domain was analyzed, classifying the studies as low, uncertain, or high risk.

### Data synthesis and statistical analyses

Continuous data were analyzed taking into account the arithmetic mean and standard deviation. In addition, for dichotomous data, relative risks (RR) with a 95% confidence interval (CI) were calculated. A fixed effects model was used through the Mantel-Haenszel method for the analysis. The effect of the intervention is presented as mean differences (MD) and RR with a 95%CI. Results were considered statistically significant if p-value was <0.05. The heterogeneity of the studies was assessed through the Pearson’s chi-square test (χ²).

## RESULTS

A total of 2,266 articles were identified in different databases, which were stored in the Rayyan tool. In the identification phase, a total of 877 duplicate articles were eliminated. Following the removal of duplicates, 1,389 articles were reviewed by two team members individually. After resolving conflicts between three members of the team, 46 articles remained to be analyzed in full text. Two of the items failed to be recovered. Of the rest, 24 were excluded due to the type of erroneous design, erroneous variable, and erroneous outcome, leaving a total of 21 articles, five of them were clinical trials and 16 were cohort studies. Data were included as study characteristics considering the name of the author, the country in which it was conducted, the year of the study, the type of research carried out, the total number of patients, and the distribution of patients according to gender, and gastric surgery, either bypass or sleeve. Thus, studies from countries such as Poland, Finland, France, Switzerland, China, Iran, Italy, the United States, Germany, Spain, and the Netherlands were included. Randomized clinical trial (n=5) and cohort (n=16) type articles were entered into the main study. Of the 21 articles entered, a total of 6,552 patients were registered, of which 1,719 were men and 4,833 were women. In addition, of the total number of patients, 3,267 were operated by gastric bypass while 3,361 were operated by gastric sleeve (Table 1).

**Table 1:** Studies included in the review.

	Author	Country	Year of study	Type of study	Total number of patients included	Patients according to sex		Patients operated with gastric bypass	Patients operated with gastric sleeve
						Male	Female		
1	Paluszkiewicz et al.^25^	Poland	2012	RCT	72	23	49	36	36
2	Helmiö et al.^11^	Finland	2014	RCT	238	157	81	171	121
3	Catheline et al.^7^	France	2019	RCT	277	39	238	91	186
4	Peterli et al.^27^	Switzerland	2018	RCT	225	69	156	113	112
5	Zhang et al.^40^	China	2014	RCT	64	38	26	32	32
6	Barzin et al.^3^	Iran	2016	Cohort	513	107	406	137	376
7	Le Foll et al.^14^	France	2020	Cohort	120	0	120	50	70
8	Perrone et al.^26^	Italy	2017	Cohort	304	94	210	142	162
9	Menguer et al.^19^	USA	2017	Cohort	102	25	77	63	39
10	Otto et al.^23^	Germany	2015	Cohort	173	51	122	127	46
11	Zhang et al.^39^	USA	2012	Cohort	558	133	425	358	200
12	Calvo et al.^6^	Spain	2020	Cohort	329	91	238	164	165
13	Fiorani et al.^10^	Germany	2020	Cohort	43	6	37	32	11
14	Jiménez et al.^12^	Spain	2019	Cohort	504	141	363	390	134
15	Lager et al.^13^	USA	2018	Cohort	714	151	563	380	334
16	Ramona et al.^29^	Netherlands	2015	Cohort	65	9	54	20	45
17	Du et al.^8^	China	2016	Cohort	126	42	84	63	63
18	Toolabi et al.^35^	Iran	´2021	Cohort	1146	249	897	396	750
19	Thereaux et al.^34^	France	2014	Cohort	359	101	258	285	74
20	Nguyen et al.^21^	USA	2018	Cohort	197	128	69	111	86
21	Barzin et al.^4^	Iran	2017	Cohort	425	65	360	106	319

RCT: randomized clinical trial.

### Methodological appraisal and risk of bias assessment

The general bias for clinical trial studies was low risk. Individually, three of the studies were categorized as low risk of bias and two as high; one theme presented a high risk in the randomization process domain and another in the measurement outcome and selection of reported outcomes. For the cohort studies, the comparability domain was the most affected. The result domain was low risk.

### Outcomes

#### Percentage of excess weight loss

All articles included percentage of excess weight loss outcome. Total data synthesis resulted in a mean deviation of -10.02 and a 95%CI of -10.14; -9.90. However, these data must be taken with caution due to the high heterogeneity (I2 of 100%).

#### Resolution of comorbidities

Four clinical trials and nine cohorts were selected for the resolution of comorbidities outcome; totaling 13 articles included for the meta-analysis, which was done individually for each of the comorbidities: DM2, AH, and DLP.

#### Diabetes mellitus type 2

The results of the total synthesis of the 13 articles mentioned above showed an RR of 0.83 with a 95%CI of 0.77–0.90 for DM. These data should also be considered with caution in clinical practice due to the high heterogeneity (I2 of 63%).

#### Arterial hypertension

The total synthesis of the 13 articles resulted in an RR of 0.77 with a 95%CI of 0.69–0.84 concerning AH. Even so, care must be taken in the use of these data in patients, due to the high heterogeneity (I2 of 78%).

#### Dyslipidemia

Of the same 13 articles, an RR of 0.83 was obtained with a 95%CI of 0.77–0.90 for DLP. It is important as well to assume this result with care because of its high heterogeneity (I2 of 74%).

#### Postoperative complications

A total of ten articles that presented results for postoperative complications outcome were included. The results of the total synthesis of these ten articles showed an RR of 0.73 with a 95%CI of 0.63–0.83, and a low heterogeneity (I2 of 16%).

Complications were divided according to their severity into major and minor, and four subgroups were assigned to this type of division.

Two subgroups reported results of major complications. The first consisted of three clinical trial studies that reported major complication events; an RR of 0.79 with a 95%CI of 0.27–2.35 was obtained, a non-statistically significant result; and a heterogeneity with I2 of 47%. The second subgroup was made up of two cohort studies, showing an RR of 0.73 with a 95%CI of 0.39–1.35, which was not statistically significant either; and presented a heterogeneity with I2 of 0%. On the other hand, the third and fourth subgroups reported results of minor complications. The third subgroup was composed of three clinical trial studies that reported minor complication events, showing an RR of 0.63 with a 95%CI of 0.33–1.18, with a statistically insignificant result; and low heterogeneity (I2 of 0%). The fourth subgroup was made up of a cohort study, with an RR of 1.20 and a 95%CI of 0.33–4.28 obtained, a result not statistically significant.

Complications were also divided according to the time of appearance, in early and late, and four subgroups were assigned to this type of division. The first two subgroups reported results of early complications. One subgroup consisted of two clinical trials showing an RR of 0.36 with a 95%CI of 0.15–0.89, and low heterogeneity (I2 of 0%). The second subgroup was made up of two cohort studies; an RR of 0.52 with a 95%CI of 0.38–0.71 was obtained, and the heterogeneity was low (I2 of 0%). Conversely, the third and fourth subgroups were composed of studies that showed late complications. Two clinical trial studies were assigned to the third subgroup; a RR of 0.67 was shown with a 95%CI of 0.38–1.16, a non-statistically significant result, and a heterogeneity with I2 of 63%. The fourth subgroup was made up of three cohorts, with an RR of 0.89 and a 95%CI of 0.75–1.06, a result that is not statistically significant either, and low heterogeneity (I2 of 0%).

#### Operative time

Seven studies were selected for this operative time outcome. The total synthesis of the data resulted in an MD of -35.76 with a 95%CI of -37.28; -34.24. We must observe this result with caution when extrapolating it into clinical practice due to the high heterogeneity (I2 of 100%).

#### Hospital stays

A total of seven articles were included in the hospital stays outcome. The total synthesis of the data obtained as a result was an MD of 0.15 with a 95%CI of -0.11–0.40, being a non-statistically significant result; heterogeneity was high (I2 of 84%).

#### Quality of life

Five articles were included — three clinical trials and two cohorts — in the quality of life outcome. The total synthesis of articles resulted in an MD of -0.37 with a 95%CI of -0.48; -0.26 and heterogeneity with I2 of 96%. Two scales were used to measure this outcome, the SF-36 (Short Form Health Survey) and the MAQQ II (Moorehead-Ardelt Quality of Life Questionnaire II)^16^.

## DISCUSSION

For a long time, RYLGB was considered the gold standard for the surgical treatment of morbid obesity; however, LSG has recently become more popular, being the second most used technique for the surgical treatment of obesity worldwide. While the LSG is a restrictive procedure, the RYLGB is a restrictive and malabsorptive procedure^16,17,29^.

This research study had excess weight loss as its main outcome, as in most studies that compare the efficacy of both surgical techniques, since this is the main objective sought when submitting a patient to bariatric surgery. In this study, it was found that LSG is significantly more effective than RYLGB in weight loss, probably because these surgical techniques have different mechanisms. Wang et al. in a systematic review reported that there is a higher percentage of excess weight loss in patients undergoing RYLGB than LSG^36^. The difference in the results may be attributed to the fact that they reported the excess weight lost only in the first year, while in the present study, both short- and long-term percentage values were considered.

Bariatric surgery enables obese patients to resolve comorbidities. Three main comorbidities were repeated in the studies that evaluated this outcome: AH, DM2, and DLP^33^. The resolution of this comorbidity is explained by the decrease in caloric intake, weight loss, malabsorption of carbohydrates and fats, and alterations in the release of hormones, including glucagon-like peptide 1 and ghrelin^41^. McTigue et al. reported that RYLGB was associated with more persistent improvements in glycemic control and 25% lower relapse rates of DM2 compared to LSG^18^. It is necessary to take into account in future studies not only the remission of DM2 but also its persistence over time. A similar result was obtained in the resolution of AH — while clinical trials do not show a statistically significant difference, cohort studies reveal results in favor of LSG^31^.

Remission of AH is defined as maintaining a blood pressure value <140/90 mmHg without the need for antihypertensive drugs; bariatric surgery produces a rapid antihypertensive effect in relation to other comorbidities^31^. This improvement is attributed to hemodynamic changes, decreased intra-abdominal pressure associated with weight loss, increased renal reabsorption of sodium, decreased arterial stiffness influenced by inflammation, and intestinal hormones^5,9^.

Resolution of DLP was slightly greater in LSG than in RYLGB, even though there was no statistical significance in the included clinical trials. This resolution was expressed in the normalization of triglyceride, low-density lipoprotein (LDL), high-density lipoprotein (HDL), and cholesterol values, as well as the absence of pharmacological use for treatment. The mechanism by which bariatric surgery promotes the resolution of DLP is through the redistribution of adipose tissue, endocrine changes, effect on inflammatory markers, metabolism of intestinal hormones, and hepatic lipid lipoproteins. Bariatric surgery is an effective treatment for severe obesity and resolution of coexisting comorbidities^2,10,14,32^.

Postoperative complications are divided into subgroups depending on the time of presentation, early and late; most studies take a cut-off point of two to three weeks depending on the study, or according to its severity, in major and minor with the Clavien-Dindo classification^15^. The most common early complications reported in the studies were leaks, fistulas, intestinal perforation, intraperitoneal hemorrhage, surgical site infection, intraperitoneal abscess, and intestinal obstruction. On the other hand, the most frequently mentioned late complications in the studies were ulcer, stenosis, vomiting, anemia, bile reflux, gastroesophageal reflux (GERD), and malnutrition. In this study, there was no statistical significance in the appearance of late complications between RYLGB and LSG, whereas there were fewer early complications in patients undergoing LSG than RYLGB. Osland et al. and Zhao et al. reported a lower rate of early complications in patients undergoing laparoscopic gastric mobilization (LGM) and a non-statistically significant result in late complications. In addition, there was no statistical significance in the appearance of major and minor complications between both surgical techniques^22,41^. Among the major complications, studies have included gastric leak, digestive fistulas, intra-abdominal bleeding, aspiration pneumonia, incarcerated incisional hernia, gastric stenosis, and deep vein thrombosis. Regarding minor complications, they have included wound infection, pain, diarrhea, dehydration, nausea, vomiting, urinary tract infection, and difficulty eating. In the study by Ali et al., the results were that both RYLGB and LSG surgical techniques were effective and safe bariatric procedures with a similar incidence of major and minor complications^1^.

There was no statistical significance in the hospital stay for both surgical techniques, even though the studies did not describe the hospital discharge criteria. On the contrary, the operative time was shorter for the LSG than for the RYGBP. This can be explained since technically the LSG is easier and faster to perform compared to the RYLGB.

Quality of life was measured using two different scales, the SF-36 and the MAQQ II, the latter implemented by the Bariatric Analysis and Reporting Outcome System (BAROS) study, which aimed to standardize the outcomes studied in surgery bariatric^16^. For the SF-36 scale, there was no statistical significance between both surgical techniques. On the contrary, using the MAQQ II scale, which is more specific for bariatric surgery, it was found that LSG is slightly more effective than RYLGB in improving the quality of life of postoperative bariatric surgery patients.

This meta-analysis study has some limitations that must be addressed. In the first place, of the 21 articles chosen, only five were clinical trials; since the majority are cohorts, they have less scientific evidence than clinical trials. Secondly, we must recognize that, although we have not considered the recurrence of obesity after bariatric surgery in the present research, this outcome should be included in future studies. There are currently few non-randomized trial studies on the tendency to regain weight after LSG 3–5 years after surgery^9^. This is a general phenomenon after bariatric surgery and is not specifically related to LGM alone. However, the included articles did not analyze this outcome, and one of the reasons is due to the follow-up time, therefore, more studies are still needed to analyze this important result. Thirdly, the heterogeneity of most of the outcomes was high, a fact that must be considered before extrapolating these results in clinical practice. This heterogeneity can be explained by the different basic characteristics of the patients included in each study, the different level surgical and postoperative care of the different hospitals. Fourthly, the length of follow-up was different in the included studies, which may be another limitation. Fifthly, even though all included studies were considered for the main outcome, not all of them were included for secondary outcomes, since some did not include these outcomes in their analysis. Sixthly, some studies had a small sample population, which may be also a limitation.

Finally, we only included studies in Spanish and English, limiting the inclusion of any study in another language that could have provided relevant data and a larger sample for the study.

## CONCLUSIONS

This meta-analysis demonstrated that LSG could be more effective than RYLGB in reducing the percentage of excess weight, fewer postoperative complications, operative time, hospital stay, and improvement in quality of life and resolution of comorbidities.
